# Longitudinal multimodal MRI analysis of lecanemab treatment in mild cognitive impairment: a pilot study of structural, perfusion, and microstructural changes

**DOI:** 10.3389/fnagi.2025.1651596

**Published:** 2025-08-26

**Authors:** Toshiya Takahashi, Dinh Ha Duy Thuy, Shingo Takenaka, Sayaka Ono, Maya Fukui, Yasushi Okada, Tomohiko Asada, Kan Niimi, Kaku Kimura, Akio Ikeda, Ryosuke Takahashi, Riki Matsumoto, Hidenao Fukuyama

**Affiliations:** ^1^Department of Neurology, Graduate School of Medicine, Kyoto University, Kyoto, Japan; ^2^Human Brain Research Center, Kyoto University Graduate School of Medicine, Kyoto, Japan; ^3^Department of Radiology, Yasu City Hospital, Yasu, Japan; ^4^Department of Rehabilitation, Yasu City Hospital, Yasu, Japan; ^5^Department of Neurology, Shiga Psychiatric Medical Center, Kusatsu, Japan; ^6^Department of Neurology, Yasu City Hospital, Yasu, Japan; ^7^Department of Epilepsy, Movement Disorders and Physiology, Kyoto University Graduate School of Medicine, Kyoto, Japan

**Keywords:** lecanemab, mild cognitive impairment (MCI), longitudinal MRI, cerebral blood flow (CBF), microstructure, hippocampus, posterior cingulate cortex, precuneus

## Abstract

**Background:**

Lecanemab, a monoclonal antibody targeting soluble amyloid-β protofibrils, has demonstrated efficacy in reducing amyloid burden in patients with mild cognitive impairment (MCI). However, its effects on brain structure, cerebral perfusion, gray matter microstructure and white matter microstructure remain unclear.

**Methods:**

This exploratory longitudinal study aimed to evaluate changes in brain volume, cerebral blood flow (CBF), and diffusion tensor imaging (DTI) measures over a 12-month treatment period in 8 patients with MCI receiving biweekly lecanemab infusions. MRI scans were acquired at baseline and at 6, 9, and 12 months using three-dimensional T1-weighted, pseudo-continuous arterial spin labeling (pCASL), and DTI sequences. Changes in whole-brain and regional indices were assessed using the Wilcoxon signed-rank test.

**Results:**

Compared to baseline, brain volume showed significant reductions at all follow-up points across all examined regions, including the whole brain, hippocampus, posterior cingulate cortex, and precuneus. CBF remained stable throughout the observation period in both global and regional analyses. Both fractional anisotropy (FA) and mean diffusivity (MD) showed significant deterioration at the whole-brain level. However, in the hippocampus, left precuneus and cingulum (cingulate gyrus), MD increased significantly at several timepoints, whereas FA remained relatively preserved, suggesting localized preservation of microstructural integrity. Neuropsychological test scores remained stable over time, with no significant deterioration observed across MMSE-J, MoCA-J, CDR-SB, or ADAS-J Cog scores. In parallel, cerebrospinal fluid biomarkers showed significant improvements in Aβ42, Aβ42/40 ratio, and p-tau181 at 6 and 12 months.

**Conclusion:**

These findings suggest that lecanemab may help maintain cerebral perfusion and partially preserve gray matter microstructure and white matter integrity during the early course of treatment in patients with MCI, despite concurrent volumetric and microstructural changes. Multimodal MRI may contribute to monitoring treatment response in patients with MCI receiving lecanemab.

## 1 Introduction

Alzheimer’s disease (AD) is a progressive neurodegenerative disorder characterized by the accumulation of amyloid-β (Aβ) plaques and abnormal hyperphosphorylation of tau protein, ultimately leading to synaptic dysfunction, brain atrophy, and cognitive decline (Bondi et al., 2017; De Wilde et al., 2016; Double et al., 1996). The accumulation of Aβ in the brain is believed to play a central role in AD pathogenesis and therapeutic agents targeting this process have been developed in recent years with the aim of slowing disease progression (Perneczky et al., 2024; Sims et al., 2023; Vitek et al., 2023). Among these, lecanemab, a humanized monoclonal antibody that selectively binds to soluble Aβ protofibrils, has demonstrated efficacy in reducing amyloid burden and slowing cognitive decline in patients with mild cognitive impairment (MCI) in previous clinical trials (van Dyck et al., 2023; Vitek et al., 2023).

Recent advances in MRI techniques provide a non-invasive, multimodal approach to assessing both macrostructural and microstructural brain changes. Structural MRI-based volumetric analyses can identify global and regional brain atrophy (Buckner et al., 2005; Frisoni et al., 2010) while diffusion tensor imaging (DTI) measures, such as fractional anisotrophy (FA) and mean diffusion (MD), can capture subtle microstructural alterations (Alexander et al., 2007; Amlien and Fjell, 2014; Chen et al., 2023). Furthermore, arterial spin labeling (ASL) of MRI enables the quantification of cerebral blood flow (CBF), offering valuable insights into vascular and metabolic brain function (Alsop et al., 2015; Dolui et al., 2020). Growing evidence indicates that changes in gray matter microstructure—particularly increased MD and decreased FA—are associated with reductions in regional CBF along the AD continuum, suggesting a close relationship between cerebral perfusion and tissue integrity (Niu et al., 2023). Consistently, diffusion MRI studies have also reported associations between regional amyloid burden and microstructural alterations in both white and gray matter (Collij et al., 2021).

These MRI techniques are widely used to monitor disease progression across the AD continuum and to evaluate the effects of anti-amyloid therapies, such as lecanemab. In line with these applications, a previous report showed volume reductions in the whole brain and hippocampus at 6, 9, and 12 months during lecanemab treatment (Swanson et al., 2021). While lecanemab has been shown to effectively reduce amyloid burden (Swanson et al., 2021; van Dyck et al., 2023), it remains unclear whether lecanemab affects other structural and functional imaging markers, such as gray matter microstructure, white matter microstructure and cerebral perfusion, as well as regional volumes of key areas including the posterior cingulate cortex and precuneus, which are central hubs of the default mode network and have been shown to exhibit reduced CBF during the prodromal stages of Alzheimer’s disease (Alsop et al., 2010; Binnewijzend et al., 2016; Ibrahim et al., 2021). Notably, a preclinical study showed that anti-amyloid antibody treatment induced vascular amyloid clearance, followed by restoration of vascular morphology, thereby supporting a vascular amyloid clearance model of ARIA (Zago et al., 2013), and we hypothesized that clearance of amyloid from both the vasculature and brain parenchyma could lead to improved cerebral blood flow. Clarifying whether lecanemab can help preserve cerebral perfusion and microstructural brain integrity in MCI patients is essential to understanding its broader therapeutic impact.

This study aimed to evaluate longitudinal changes in brain volume, cerebral perfusion, gray matter microstructure and white matter microstructure in relation to cognitive performance in MCI patients treated with lecanemab, focusing on the 6- to 12-month period of treatment. In addition to whole brain analysis, we examined specific regions known to be affected early in the AD continuum, such as the hippocampus, posterior cingulate cortex, and precuneus. This study provides preliminary insights into the functional and structural effects of lecanemab in patients with MCI.

## 2 Materials and methods

### 2.1 Study design and participants

This prospective study was conducted at Yasu City Hospital. Participants were enrolled based on the following neuropsychological and cerebrospinal fluid (CSF) biomarker criteria: Mini-Mental State Examination (MMSE) score between 22 and 28, Montreal Cognitive Assessment (MoCA) score between 18 and 25, Clinical Dementia Rating-Global Score (CDR-GS) between 0.5 and 1, and Geriatric Depression Scale (GDS) score of 8 or lower. CSF biomarkers included an amyloid-β 1–42 to 1–40 ratio (Aβ42/40 ratio) of 0.67 or lower and phosphorylated tau 181 (p-tau181) of 59.0 pg/mL or higher. For a more detailed assessment of cognitive function, the CDR-Sum of Boxes (CDR-SB) and Alzheimer’s Disease Assessment Scale-Cognitive Subscale (ADAS-Cog) were also administered (Table 1).

**Table 1:** Participant demographics and baseline characteristics.

Category	Value
Sex (F/M)	6/2
Age, mean ± SD (range)	71.62 ± 6.97 (60–79)
ApoE genotype	3/3: 5, 3/4: 1, 4/4: 2
MMSE-J	25.0 ± 2.07 (22–28)
MoCA-J	19.25 ± 1.28 (18–21)
CDR-SB	2.69 ± 1.25 (1–5)
CDR-GS	0.56 ± 0.18 (0.5–1)
ADAS-J Cog	14.12 ± 5.03 (8–24)
Aβ42 (pg/mL)	713.0 ± 148.85 (551–938)
Aβ42/40 ratio	0.044 ± 0.008 (0.029–0.055)
p-tau181 (pg/mL)	91.64 ± 44.99 (51–166)

CDR, Clinical Dementia Rating; MMSE-J, Mini-Mental State Examination-Japanese version; MoCA-J, Montreal Cognitive Assessment-Japanese version; CDR-SB, Clinical Dementia Rating-Sum-of-Boxes; CDR-GS, Clinical Dementia Rating-Global Score; ADAS-J Cog, Alzheimer’s Disease Assessment Scale-Cognitive subscale-Japanese version; Aβ, amyloid-beta; p-tau181, phosphorylated tau at threonine 181.

All participants were screened using structural MRI, including T1-weighted, FLAIR, SWI, and time-of-flight MRA sequences. We excluded individuals with any evidence of large territorial infarction, intracerebral hemorrhage, intracranial mass lesions, or significant asymmetry or stenosis in the internal carotid artery system.

The study included 8 participants (6 females and 2 males) aged between 60 and 79 years. Each participant received lecanemab infusions every 2 weeks, and evaluations were conducted between 6 months and 1 year after the initiation of treatment.

### 2.2 Ethical considerations

This prospective observational study was approved by the Ethics Committee of Yasu City Hospital. As the study involved only the analysis of data obtained during routine clinical care, and posed no additional risk or burden to participants, the requirement for written informed consent was waived in accordance with institutional ethical guidelines.

### 2.3 MRI acquisition

All MRI data were collected using a 3 T MRI system (Lumina; Siemens, Erlangen, Germany), equipped with a 32-channel head coil. Structural 3D T1-weighted images were acquired using three-dimensional magnetization-prepared rapid gradient-echo (3D-MPRAGE) sequences with the following parameters: repetition time (TR) = 1,240 ms, echo time (TE) = 2.88 ms, inversion time (TI) = 900 ms, flip angle (FA) = 10°, field of view (FOV) = 256 × 256 mm, resolution 1 × 1 × 2 mm^3^. ASL images were acquired by employing 3D fast spin echo pseudo-continuous ASL (pCASL) sequence with acquisition parameters: TR = 4,500 ms, TE = 21.80 ms, FA = 120°, voxel size = 1.8 × 1.8 × 5 mm^3^, NEX = 3 and post label delay = 2,000 ms. For the WM microstructural evaluation, a standard DTI protocol was performed with the following parameters TR = 10,800 ms, TE = 108 ms, voxel size = 2 × 2 × 2 mm^3^, gap = 0, 30 diffusion directions with b = 1,500 s/mm^2^.

All participants underwent MRI scans before the initiation of lecanemab treatment, with follow-up scans conducted at the 13th (6 months), 20th (9 months), and 26th (1 year) administrations after treatment initiation. At each time point, imaging was performed using 3D T1-weighted images, pCASL, and DTI sequences to analyze longitudinal changes in brain volume, CBF, gray matter microstructure and white matter microstructure.

### 2.4 MRI processing

All MRI data in DICOM format were converted to NIfTI format using dcm2niix (version 1.0.20240202) (Li et al., 2016). T1-weighted images underwent bias field correction, skull stripping, and tissue segmentation using the fsl_anat tool from FSL (version 6.0.7.13) (Smith et al., 2004). ASL images were processed using the BASIL module (oxford_asl) based on a Bayesian inference model (Chappell et al., 2009), and CBF maps were generated.

DTI data were processed using topup (Andersson et al., 2003), eddy (Andersson et al., 2018; Andersson et al., 2017; Andersson et al., 2016; Andersson and Sotiropoulos, 2016), and DTIFIT, all of which are part of FSL, to correct for magnetic field and eddy current distortions and to compute voxel-wise diffusion measures such as FA and MD. DTI analysis was performed using the standard pipeline provided by FSL.

To enable integrated analysis within the same spatial framework, ASL and DTI data were accurately registered to FreeSurfer space using bbregister from FreeSurfer (version 8.0.0). Longitudinal processing in FreeSurfer was applied to improve within-subject consistency across timepoints. Brain volume measures were obtained from FreeSurfer (Fischl et al., 2002), while CBF, FA, and MD values derived from FSL were resampled into the same space. ROI analyses targeted the hippocampus, posterior cingulate cortex and precuneus, which are key regions along the Alzheimer’s disease continuum and were defined anatomically using FreeSurfer.

### 2.5 Statistical analysis

#### 2.5.1 Imaging marker statistical analysis

Longitudinal changes in brain volume, CBF, FA, and MD were evaluated at baseline, 6 months, 9 months, and 12 months. Values were extracted from the whole brain and ROIs, including the hippocampus, posterior cingulate cortex, and precuneus. Statistical analyses between baseline and each follow-up time point were conducted in R (version 4.4.2), using the Wilcoxon signed-rank test to evaluate changes at both whole-brain and ROI levels.

#### 2.5.2 Neuropsychological and CSF biomarker statistical analysis

Changes in neuropsychological test scores and CSF biomarkers between baseline and each follow-up time point (6 months, 9 months, and 12 months) were similarly evaluated using the Wilcoxon signed-rank test. The variables included in the statistical analysis were ADAS-J Cog, MMSE-J, MoCA-J, CDR-SB, Aβ42, Aβ42/40 ratio, and p-tau181.

#### 2.5.3 Statistical considerations

As this study is exploratory with a small sample size, statistical analyses of imaging markers, neuropsychological tests, and CSF biomarkers report uncorrected *p*-values without adjustment for multiple comparisons.

## 3 Results

### 3.1 Neuropsychological measures

**Figure 1:**
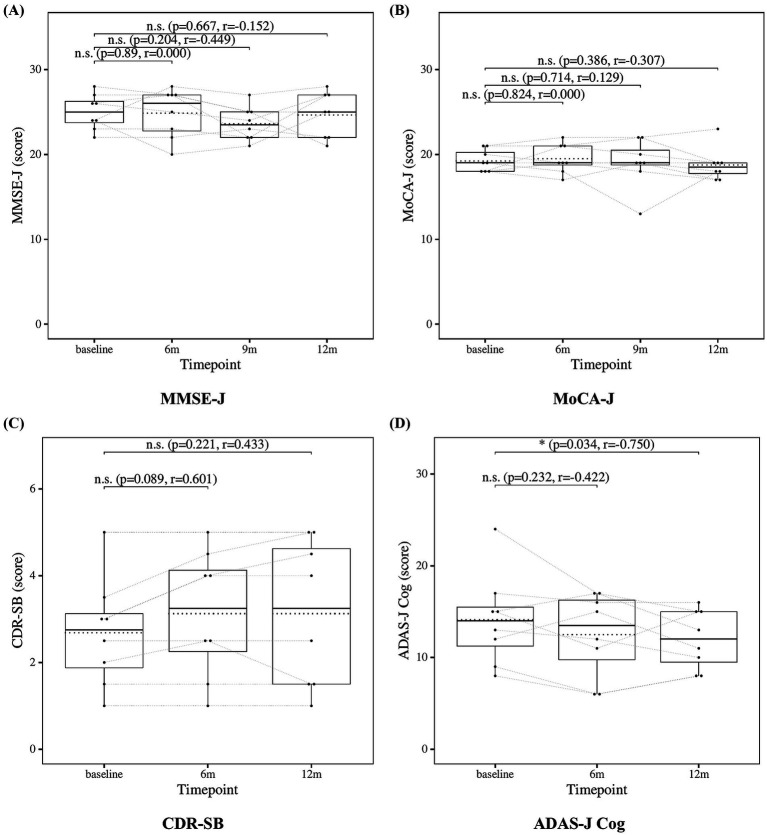
Change from baseline in cognitive test scores. **(A)** MMSE-J and **(B)** MoCA-J results at four time points (baseline, 6, 9, and 12 months). **(C)** CDR-SB and **(D)** ADAS-J Cog results at three time points (baseline, 6, and 12 months). Solid lines indicate medians; dotted lines indicate means. Statistical significance was assessed by Wilcoxon signed-rank tests: *p* < 0.05, *p* < 0.01, and *p* < 0.001; n.s., not significant. Effect sizes were reported as *r* values.

The results of the statistical analysis of neuropsychological tests are presented in Figure 1. No significant changes were observed in the neuropsychological test scores, except for ADAS-J Cog at the 12-month time point, which showed a significant improvement.

### 3.2 CSF biomarkers measures

**Figure 2:**
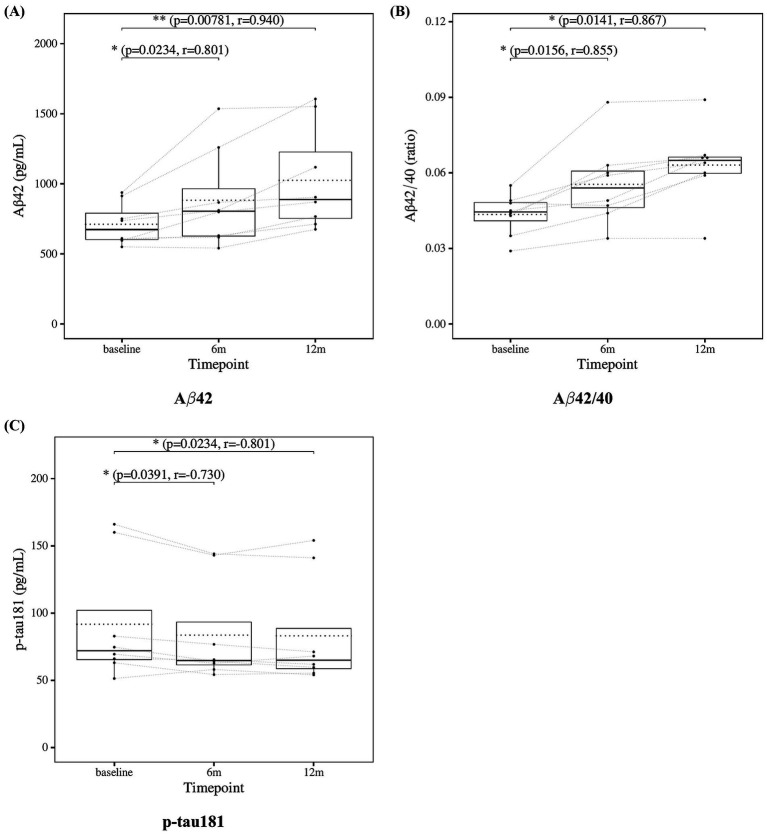
Change from baseline in cerebrospinal fluid (CSF) biomarker levels. **(A)** Aβ42, **(B)** Aβ42/40 ratio, and **(C)** p-tau181 results at three time points (baseline, 6, and 12 months). Solid lines indicated medians; dotted lines indicated means. Statistical significance was assessed by Wilcoxon signed-rank tests: *p* < 0.05, *p* < 0.01, and *p* < 0.001; n.s., not significant. Effect sizes were reported as *r* values.

The results of the statistical analysis of CSF biomarkers are presented in Figure 2. Significant improvements were observed in Aβ42, Aβ42/40 ratio, and p-tau181 at both the 6-month and 12-month time points.

### 3.3 Whole-brain measures

**Figure 3:**
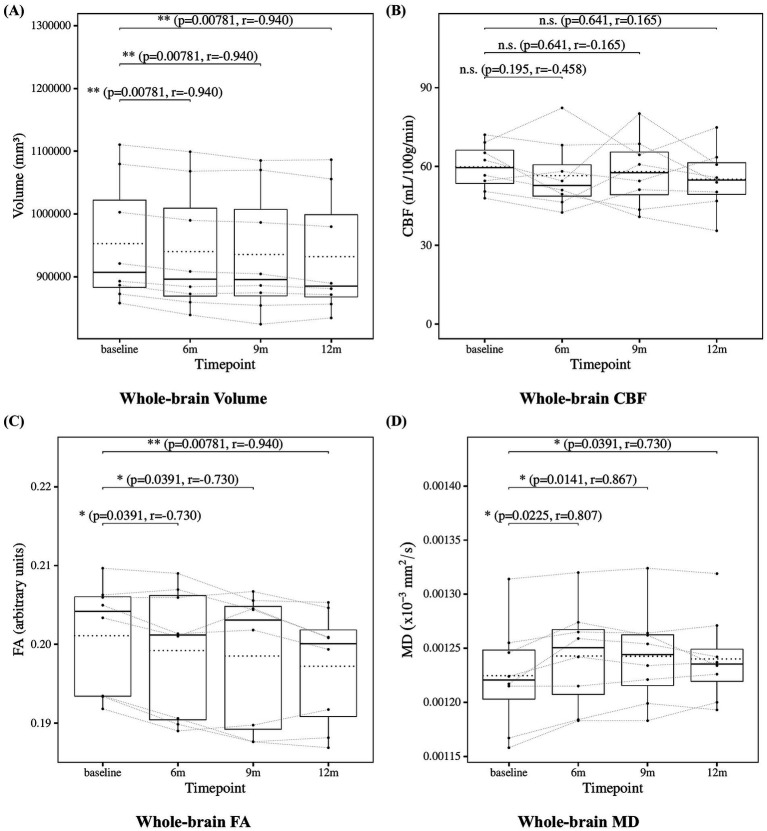
Change from baseline in whole-brain measures. **(A)** Whole-brain volume, **(B)** whole-brain CBF, **(C)** whole-brain FA, and **(D)** whole-brain MD results at four time points (baseline, 6, 9, and 12 months). Solid lines indicated medians; dotted lines indicated means. Statistical significance was assessed by Wilcoxon signed-rank tests: *p* < 0.05, *p* < 0.01, and *p* < 0.001; n.s., not significant. Effect sizes were reported as *r* values.

The results of the statistical analysis of whole-brain measures are presented in Figure 3. Whole-brain volume showed a significant decrease at 6, 9, and 12 months compared to baseline. Whole-brain CBF did not show any significant changes at any follow-up time point. Whole-brain FA showed a significant decrease, and whole-brain MD showed a significant increase at 6, 9, and 12 months compared to baseline.

### 3.4 Hippocampal measures

**Figure 4:**
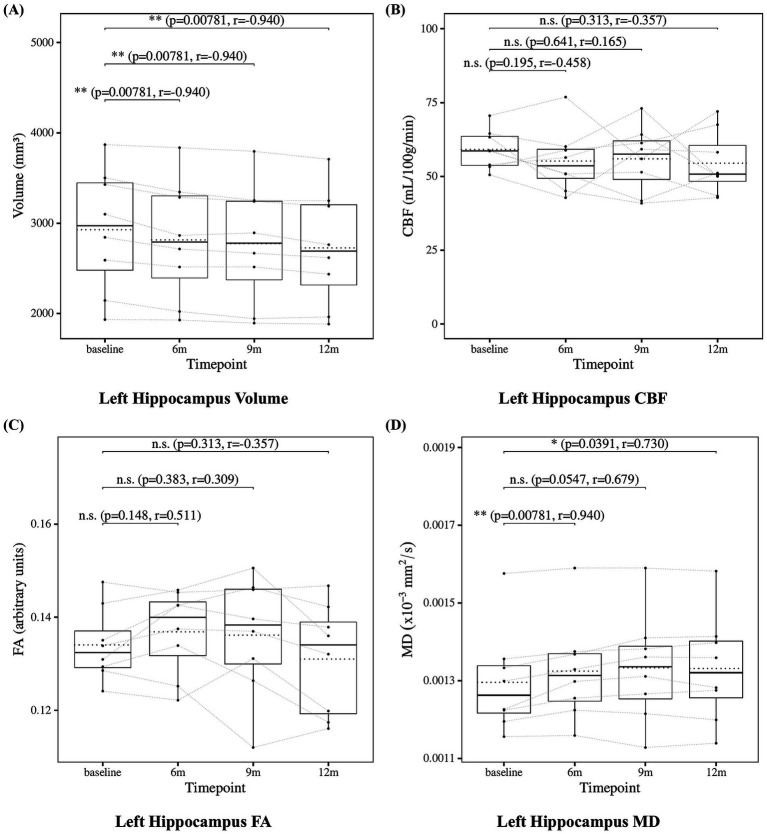
Change from baseline in left hippocampal measures. **(A)** Left hippocampus volume, **(B)** left hippocampus CBF, **(C)** left hippocampus FA, and **(D)** left hippocampus MD results at four time points (baseline, 6, 9, and 12 months). Solid lines indicate medians; dotted lines indicate means. Statistical significance was assessed by Wilcoxon signed-rank tests: *p* < 0.05, *p* < 0.01, and *p* < 0.001; n.s., not significant. Effect sizes were reported as *r* values.

**Figure 5:**
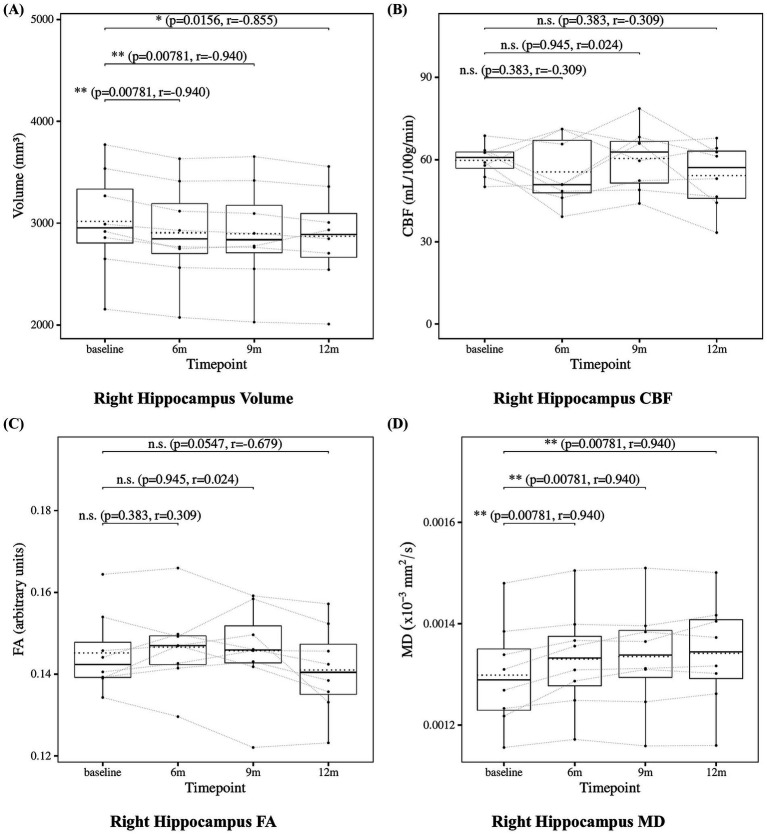
Change from baseline in right hippocampal measures. **(A)** Right hippocampus volume, **(B)** right hippocampus CBF, **(C)** right hippocampus FA, and **(D)** right hippocampus MD results at four time points (baseline, 6, 9, and 12 months). Solid lines indicated medians; dotted lines indicated means. Statistical significance was assessed by Wilcoxon signed-rank tests: *p* < 0.05, *p* < 0.01, and *p* < 0.001; n.s., not significant. Effect sizes were reported as *r* values.

The results of the statistical analysis of hippocampal measures are presented in Figure 4 for the left hippocampus and Figure 5 for the right hippocampus. Hippocampal volume showed a significant decrease at 6, 9, and 12 months compared to baseline. Hippocampal CBF showed no significant changes at 6, 9, or 12 months. Hippocampal FA also showed no significant changes at 6, 9, or 12 months. Although the decrease in right hippocampal FA at 12 months did not reach statistical significance (*p* = 0.0547), the effect size was large (*r* = −0.679), suggesting a meaningful trend of microstructural deterioration. Hippocampal MD showed a significant increase at 6, 9 and 12 months except for the left hippocampus at 9 months. Although the increase at 9 months in left hippocampal did not reach statistical significance (*p* = 0.0547), the effect size was large (*r* = 0.679), suggesting a consistent and meaningful trend of microstructural deterioration over time.

### 3.5 Posterior cingulate cortex measures

**Figure 6:**
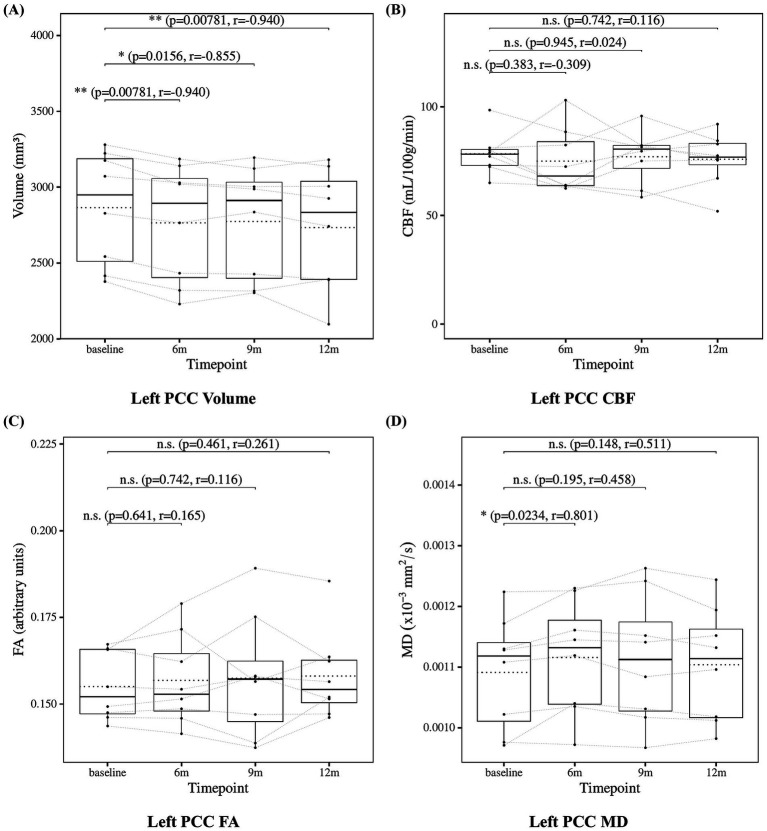
Change from baseline in left posterior cingulate cortex (PCC) measures. **(A)** Left PCC volume, **(B)** left PCC CBF, **(C)** left PCC FA, and **(D)** left PCC MD results at four time points (baseline, 6, 9, and 12 months). Solid lines indicated medians; dotted lines indicated means. Statistical significance was assessed by Wilcoxon signed-rank tests: *p* < 0.05, *p* < 0.01, and *p* < 0.001; n.s., not significant. Effect sizes were reported as *r* values.

**Figure 7:**
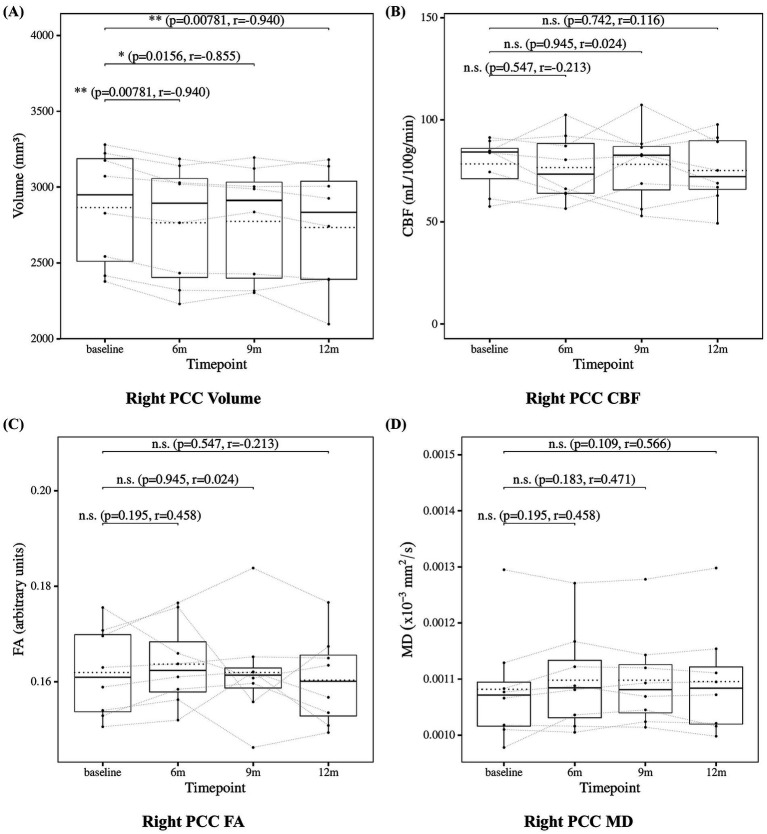
Change from baseline in right posterior cingulate cortex (PCC) measures. **(A)** Right PCC volume, **(B)** right PCC CBF, **(C)** right PCC FA, and **(D)** right PCC MD results at four time points (baseline, 6, 9, and 12 months). Solid lines indicated medians; dotted lines indicated means. Statistical significance was assessed by Wilcoxon signed-rank tests: *p* < 0.05, *p* < 0.01, and *p* < 0.001; n.s., not significant. Effect sizes were reported as *r* values.

The results of the statistical analysis of posterior cingulate cortex measures are presented in Figure 6 for the left posterior cingulate cortex and Figure 7 for the right posterior cingulate cortex. Posterior cingulate cortex volume showed a significant decrease at 6, 9, and 12 months compared to baseline. Posterior cingulate cortex CBF showed no significant changes at 6, 9, or 12 months. Posterior cingulate cortex FA also showed no significant changes at 6, 9, or 12 months. Posterior cingulate cortex MD showed no significant changes at 6, 9, or 12 months except for the left posterior cingulate cortex at 6 months, which showed a significant increase.

### 3.6 Precuneus measures

**Figure 8:**
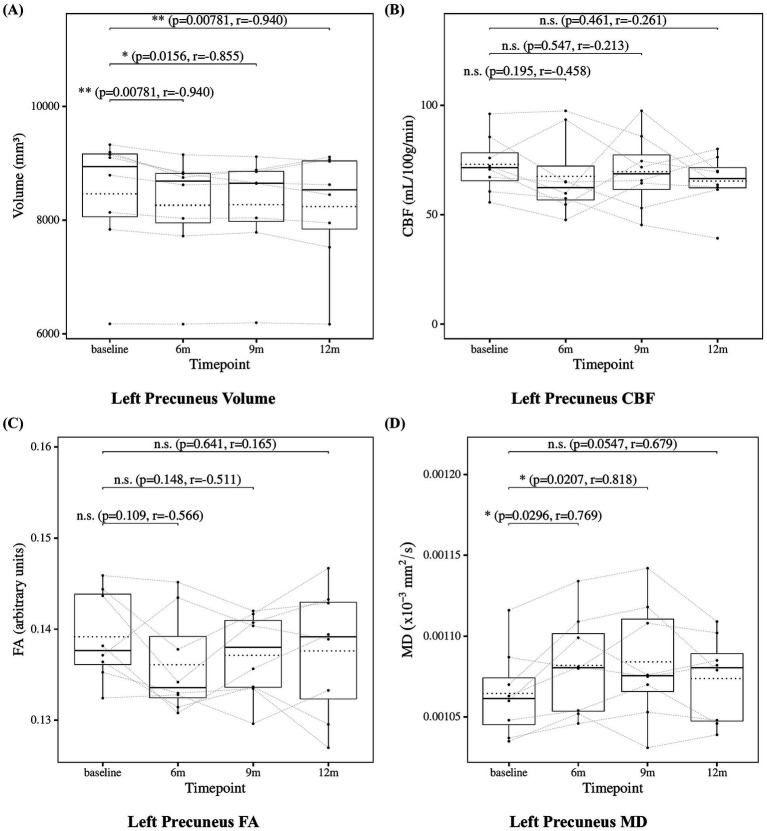
Change from baseline in left precuneus measures. **(A)** Left precuneus volume, **(B)** left precuneus CBF, **(C)** left precuneus FA, and **(D)** left precuneus MD results at four time points (baseline, 6, 9, and 12 months). Solid lines indicated medians; dotted lines indicated means. Statistical significance was assessed by Wilcoxon signed-rank tests: *p* < 0.05, *p* < 0.01, and *p* < 0.001; n.s., not significant. Effect sizes were reported as *r* values.

**Figure 9:**
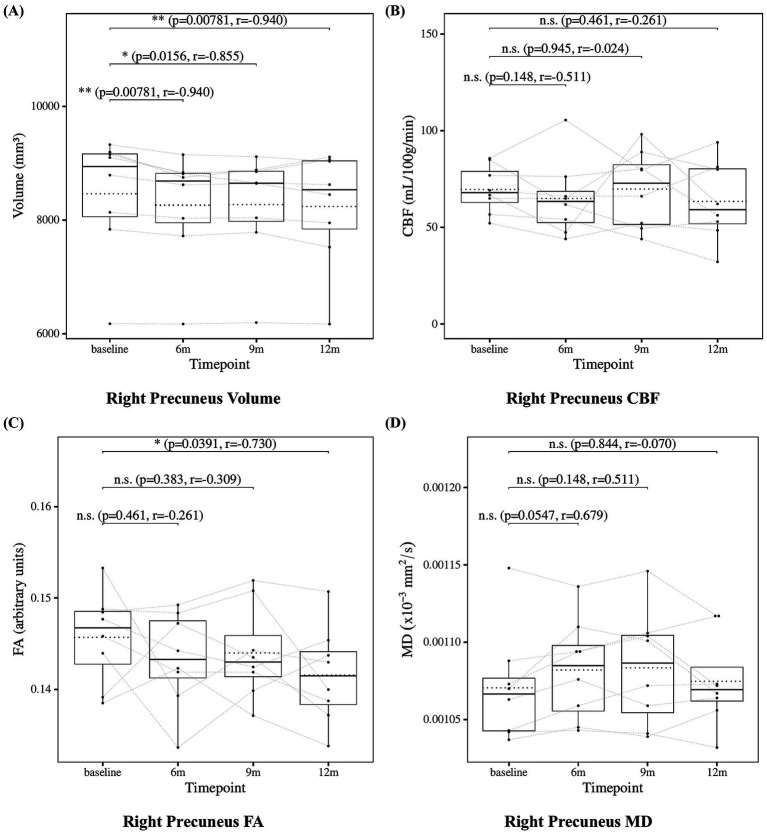
Change from baseline in right precuneus measures. **(A)** Right precuneus volume, **(B)** right precuneus CBF, **(C)** right precuneus FA, and **(D)** right precuneus MD results at four time points (baseline, 6, 9, and 12 months). Solid lines indicated medians; dotted lines indicated means. Statistical significance was assessed by Wilcoxon signed-rank tests: *p* < 0.05, *p* < 0.01, and *p* < 0.001; n.s., not significant. Effect sizes were reported as *r* values.

The results of the statistical analysis of precuneus measures are presented in Figure 8 for the left precuneus and Figure 9 for the right precuneus. Precuneus volume showed a significant decrease at 6, 9, and 12 months compared to baseline. Precuneus CBF showed no significant changes at 6, 9, or 12 months. Precuneus FA showed no significant changes at 6, 9, or 12 months except for the right precuneus at 12 months, which showed a significant decrease. Left precuneus MD showed a significant increase at 6 and 9 months. Although the increase at 12 months in left precuneus MD did not reach statistical significance (*p* = 0.0547), the effect size was large (*r* = −0.679), suggesting a consistent and meaningful trend of microstructural deterioration over time. Right precuneus MD showed no significant changes at 6, 9, or 12 months. Although the increase at 6 months in right precuneus did not reach statistical significance (*p* = 0.0547), the effect size was large (*r* = 0.679), suggesting a meaningful trend of microstructural deterioration.

### 3.7 Cingulum measures

**Figure 10:**
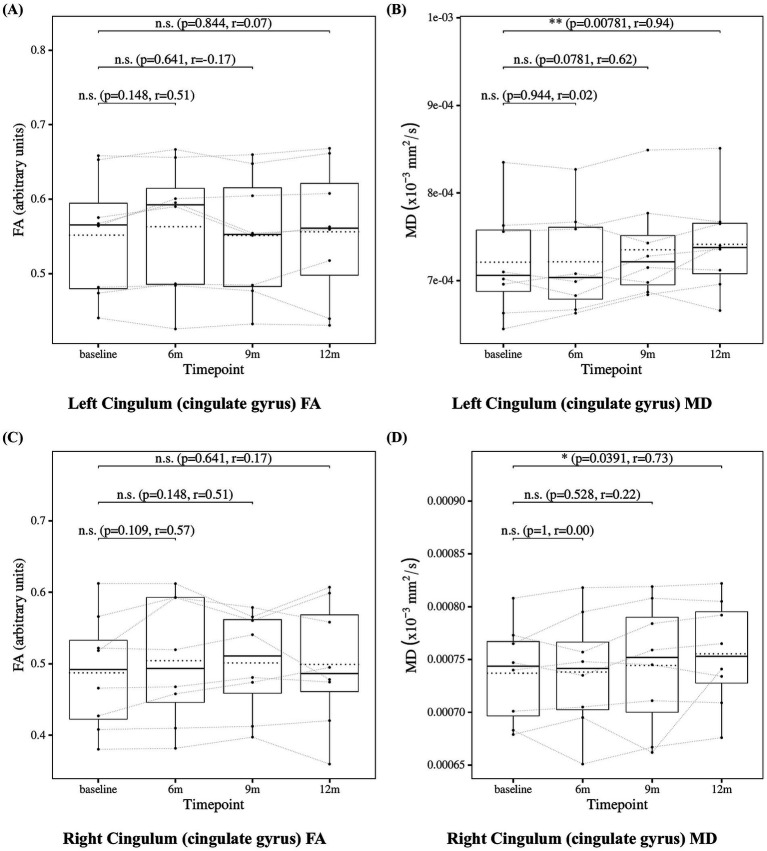
Change from baseline in cingulum (cingulate gyrus) measures. **(A)** Left cingulum (cingulate gyrus) FA and **(B)** left cingulum (cingulate gyrus) MD, **(C)** right cingulum (cingulate gyrus) FA, and **(D)** right cingulum (cingulate gyrus) MD results at four time points (baseline, 6, 9, and 12 months). Solid lines indicate medians; dotted lines indicate means. Statistical significance was assessed by Wilcoxon signed-rank tests: *p* < 0.05, *p* < 0.01, and *p* < 0.001; n.s., not significant. Effect sizes were reported as *r* values.

**Figure 11:**
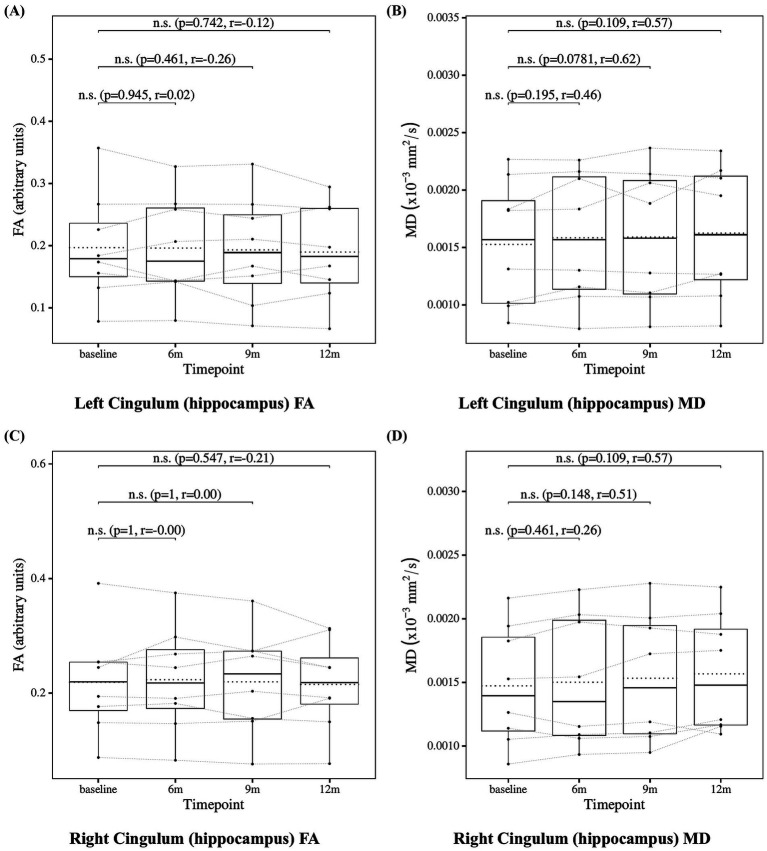
Change from baseline in cingulum (hippocampus) measures. **(A)** Left cingulum (hippocampus) FA and **(B)** left cingulum (hippocampus) MD, **(C)** right cingulum (hippocampus) FA, and **(D)** right cingulum (hippocampus) MD results at four time points (baseline, 6, 9, and 12 months). Solid lines indicate medians; dotted lines indicate means. Statistical significance was assessed by Wilcoxon signed-rank tests: *p* < 0.05, *p* < 0.01, and *p* < 0.001; n.s., not significant. Effect sizes were reported as *r* values.

To further investigate, we analyzed the cingulum bundle, a white matter tract that anatomically connects the hippocampus, posterior cingulate cortex, and precuneus. The results of the statistical analysis of cingulum measures are presented in Figure 10 for the cingulum (cingulate gyrus) and in Figure 11 for the cingulum (hippocampus), both defined according to the JHU white matter atlas from FSL. The cingulum (cingulate gyrus) showed a significant increase in MD at 12 months compared to baseline, whereas the cingulum (hippocampus) showed no significant changes. These findings may suggest progressive microstructural alterations during lecanemab treatment.

## 4 Discussion

### 4.1 Summary of longitudinal imaging and cognitive changes

In this exploratory longitudinal study of patients with MCI receiving lecanemab, we observed progressive reductions in brain volume—including whole brain, hippocampus, posterior cingulate cortex, and precuneus—over a 6-to-12-month treatment period. In contrast, CBF remained stable across all examined regions. DTI measures revealed a more heterogeneous pattern: MD increased in the whole brain, the bilateral hippocampus, the left precuneus, and the cingulum (cingulate gyrus), while FA remained relatively stable in regions closely associated with cognitive function. Notably, cognitive performance did not show significant deterioration, while significant improvements were observed in Aβ42, the Aβ42/40 ratio, and p-tau181 over the course of the study.

### 4.2 Stability of CBF under lecanemab treatment

While alterations in CBF, including both decreases and transient increases, have been reported during the MCI stage of Alzheimer’s disease, the absence of significant change in our cohort may reflect hemodynamic stability during lecanemab treatment (Alsop et al., 2010; Binnewijzend et al., 2016). This stability was observed even in regions such as the posterior cingulate cortex and precuneus, which are among the earliest to exhibit CBF reduction in the prodromal stage of Alzheimer’s disease (Alsop et al., 2010; Binnewijzend et al., 2016). These findings suggest that lecanemab may help stabilize vascular and metabolic function in the early stages of disease progression.

### 4.3 Dissociation between FA and MD trajectories

With respect to gray matter microstructure and white matter microstructure, our findings revealed a dissociation between FA and MD trajectories. FA remained relatively stable in key regions such as the hippocampus, posterior cingulate cortex, precuneus, and the cingulum (cingulate gyrus), while MD showed significant increases primarily in the bilateral hippocampus, left precuneus, and the cingulum (cingulate gyrus). This pattern is consistent with previous studies suggesting that MD may be more sensitive than FA in detecting early microstructural alterations along the Alzheimer’s disease continuum (Nir et al., 2013; Niu et al., 2023).

### 4.4 Relationship between gray matter microstructure, white matter integrity, and cognitive preservation

Previous studies have demonstrated associations between DTI measures and cognitive performance in individuals with Alzheimer’s disease and MCI (Chen et al., 2023; Nir et al., 2013). In this context, the relative preservation of FA in regions implicated in memory and higher-order cognitive functions, such as the hippocampus, posterior cingulate cortex, and precuneus, may at least partly account for the stable cognitive profiles observed in the present cohort. Although causality cannot be inferred, the partial preservation of gray matter microstructure and white matter integrity in these areas may contribute to the maintenance of cognitive function despite concurrent volume loss and MD increases. These findings emphasize the value of integrating diffusion imaging with structural and perfusion measures to better understand the multifaceted nature of disease progression and treatment response.

### 4.5 Regional patterns of structural and microstructural change

The hippocampus, precuneus, and posterior cingulate cortex are among the regions vulnerable to early alterations in Alzheimer’s disease (Alsop et al., 2010; Buckner et al., 2005; Chen et al., 2023; Frisoni et al., 2010). In the present study, all three regions showed progressive volume loss. Changes in MD were observed primarily in the hippocampus, left precuneus, and the cingulum (cingulate gyrus), where MD increased in parallel with volume reduction, while CBF and FA remained relatively stable. This discrepancy between structural degeneration and preserved perfusion or axonal integrity may represent an intermediate stage of disease progression. These findings suggest that regional assessment of volume, MD, CBF, and FA may provide sensitive markers of treatment response in MCI patients receiving lecanemab.

### 4.6 Limitations

Several limitations of this study should be acknowledged. First, the small sample size (*n* = 8) limits statistical power and increases the risk of both Type I and Type II errors. The absence of a control group further restricts the ability to isolate treatment effects from the natural course of disease progression. In addition, multiple comparisons were performed without correction due to the exploratory nature of the study, which necessitates cautious interpretation of the findings. Moreover, the sex distribution of participants was unbalanced (6 females and 2 males), which may have amplified individual variability and limited the generalizability of the findings. Finally, as all participants were recruited from a single site in Japan, potential differences related to ethnic, genetic, or environmental backgrounds should be considered when generalizing these findings to non-Asian populations. These limitations underscore the need for larger, controlled studies to validate and extend the present observations.

## 5 Conclusion

In conclusion, this exploratory study suggests that cognitive function was preserved during the first year of lecanemab treatment in patients with MCI, despite progressive structural and microstructural brain changes. No significant changes in CBF were observed in any examined regions, including the posterior cingulate cortex and precuneus—areas typically affected early in AD—suggesting that lecanemab may help maintain cerebral perfusion during the early disease stages.

In contrast, MD increased in several regions such as the hippocampus, precuneus, and the cingulum (cingulate gyrus), even as FA remained relatively stable, indicating that gray matter microstructure and white matter integrity may have been only partially and temporarily preserved. This dissociation between vascular/metabolic stability and microstructural degeneration suggests that lecanemab may help preserve perfusion and partially maintain gray matter microstructure and white matter integrity in regions including the hippocampus, posterior cingulate cortex, precuneus, and the cingulum (cingulate gyrus), even as structural decline progresses.

These findings underscore the utility of multimodal imaging in detecting early therapeutic effects and suggest that preservation of CBF and axonal integrity may be associated with maintenance of cognitive function in MCI patients receiving lecanemab.

## Data Availability

The raw clinical data supporting the conclusions of this article are not publicly available due to patient privacy and ethical restrictions. Further enquiries or requests to access the datasets should be directed to the corresponding author.
